# Soil Pore Network Complexity Changes Induced by Wetting and Drying Cycles—A Study Using X-ray Microtomography and 3D Multifractal Analyses

**DOI:** 10.3390/ijerph191710582

**Published:** 2022-08-25

**Authors:** Jocenei A. T. de Oliveira, Fábio A. M. Cássaro, Adolfo N. D. Posadas, Luiz F. Pires

**Affiliations:** 1 Laboratory of Physics Applied to Soils and Environmental Sciences, State University of Ponta Grossa, Ponta Grossa 84030-900, PR, Brazil; 2 AgriEntech Ltda., São Carlos 13560-460, SP, Brazil

**Keywords:** 3D geometric parameters, generalized fractal dimension, conservation agriculture, no-tillage system, soil structure

## Abstract

Soils are dynamic and complex systems in their natural state, which are subjected to profound changes due to management. Additionally, agricultural soils are continuously exposed to wetting and drying (W-D) cycles, which can cause modifications in the complexity of their pores. Thus, we explore how successive W-D cycles can affect the pore network of an Oxisol under contrasting managements (conventional tillage—CT, minimum tillage—MT, no tillage—NT, and secondary forest—F). The complexity of the soil pore architecture was evaluated using a 3D multifractal approach combined with lacunarity, Shannon’s entropy, and pore geometric parameters. Our results showed that the multifractal approach effectively identified and quantified the changes produced in the soil pore architecture by the W-D cycles. The lacunarity curves revealed important aspects of the modifications generated by these cycles. Samples under F, NT, and MT suffered the most significant changes. Pore connectivity and tortuosity were largely affected by the cycles in F and NT. Our findings demonstrated that the 3D geometric parameters and normalized Shannon’s entropy are complementary types of analysis. According to the adopted management, they allowed us to separate the soil into two groups according to their similarities (F and NT; CT and MT).

## 1. Introduction

Soils are considered dynamic and complex systems, whose structures are continuously transformed due to weather-related factors such as wetting, drying, freezing, and thawing. Besides natural processes, management practices can also induce changes in the soil’s pore systems. When soils under native forests are managed for agricultural purposes, the complexity of their pores is strongly altered depending on how this porous system is managed [1,2,3,4,5]. As soil quality is strictly related to its structure, the impact of management or natural processes will directly affect the architecture of the pores in the soil matrix [6,7,8].

Appropriate soil management practices are crucial for improving soil quality and crop yield. On the other hand, inadequate management damages the soil, impacting its functionality [9,10]. Thus, studies aiming to quantify the soil pore system after the adoption of different management practices are fundamental to understanding how the soil structure is modified [11,12]. Furthermore, special attention is necessary on the changes in the soil pore system at the scale of micro- and macropores, which alter the way the water moves through and is retained by the soil [13,14,15].

In addition to management practices, the soil structure is especially susceptible to changes due to the action of wetting and drying (W-D) cycles caused by natural (rainfall) and artificial (irrigation) processes. Wetting and drying cycles often cause the increase in the complexity of the pore system, as they alter the morphology and topology of soil pores. These changes contribute to modifications in the soil’s ecological functioning with impacts on water movement, soil aeration, nutrient cycling, C storage, and turnover, to cite some of them [2,16,17,18].

As the structure of the soil can be considered a non-linear system, multifractal theories have been proved to be appropriate for characterizing the soil pore system by detecting self-similarity patterns, fractional dimensions, and global information [19,20,21]. In recent years, multifractal analysis has usually been employed to quantify parameters linked to complex systems such as, for instance, their physical and thermodynamic properties [22,23,24]. Additionally to the multifractal analysis, lacunarity is another useful tool employed for outlining the heterogeneity and complexity of complex pore systems [24,25]. Lacunarity correlates the distribution of lacunes or the number of pores to the investigated scale of analysis.

However, the multifractal or lacunarity analyses are usually accomplished by investigating reconstructed high-quality 3D X-ray computed microtomography (µCT) images. The 3D analysis permits evaluating parameters such as the connectivity (C) and the tortuosity (τ) of the soil pores, as well as their degree of anisotropy (DA). The quantification of these parameters is highly relevant as they can generate relevant and non-standard results to understand how management practices and W-D cycles affect the soil structure [26,27,28,29,30,31].

A better comprehension of the impact of W-D cycles in the structure of soils submitted to contrasting management practices is of extreme relevance. Understanding how these cycles affect the 3D soil pore system’s complexity might bring new insights to observe how its structure reacts when submitted to sequences of wetting and drying cycles. Existing studies on the impact of W-D cycles on soil pore systems present only qualitative results or are limited to the analysis of just one management practice. To the best of our knowledge, there is no type of study characterizing the soil pore complexity changes induced by W-D cycles using 3D multifractal and lacunarity concepts. Thus, this study proposes using X-ray µCT high-resolution 3D images plus multifractal, lacunarity, Shannon’s entropy, and morphological analyses to understand the effect of W-D cycles in the pore system complexity of an Oxisol under contrasting management practices.

## 2. Materials and Methods

### 2.1. Sample Collection and Location

Samples of an Oxisol (Rhodic Hapludox), according to the Soil Survey Staff [32], were collected from an experimental farm of the “Instituto de Desenvolvimento Rural do Paraná”, located in the municipality of Ponta Grossa, Brazil (−25.15, −50.15, 875 m a.s.l.). Samples from this experimental field have been employed in many studies dealing with soil physical properties and computed tomography. These areas have been subjected to long-term experiments for more than 35 years. The Oxisols are the most representative soil found in Brazil, covering around 39% of the country’s area [33]. The soil samples were extracted from plots under three contrasting management practices (conventional tillage—CT, minimum tillage—MT, and no tillage—NT). An area under secondary forest (F) adjacent to these experimental plots was selected as a reference plot.

The experimental areas under CT, MT, and NT were initiated in 1981 after converting part of a secondary forest to pasture-land [34]. The soil under conventional tillage was subjected to disking at a 25 cm depth, followed by harrowing twice a year after winter and summer harvests. The soil under minimum tillage was subjected to a chisel cultivator at a depth of 25 cm, followed by narrow disking at 10 cm, and the crop residues were kept on the soil surface. The preparation of the soil under no tillage was restricted to sowing with a cutting disk. In the experimental areas, crop rotation was performed with cover crops (oats (*Avena strigosa*) or vetch (*Vicia sativa*)) or wheat (*Triticum aestivum* L.) in winter and corn (*Zea mays*) or soybean (*Glycine max*) in summer [29,30]. Commercial tillage machinery (tractors weighing around four tons) was employed in all the operations (soil and crop management, sowing, harvest, clearing, and planting seed).

Before sampling, the top 3 cm of soil was removed to facilitate collection due to root and leaf deposition, mainly in the NT and MT experimental fields. The samples were collected from the topsoil layer (0–0.10 m) using steel cylinders, c. 5 cm in diameter × c. 5 cm in height. They were sampled at the field capacity (compared to the sample soil moisture content obtained through the water retention curve) to avoid damage to soil structure during the collection, about three days after a high-intensity rainfall event. The samples were gently collected by placing the steel cylinders into the soil surface using a soil core sampler cup. After the cylinders were placed, the surrounded soil was carefully excavated for the sample cylinder extraction. The clay, silt, and sand contents in the top layer were 530 g kg^−1^ clay, 300 g kg^−1^ silt, 170 g kg^−1^ sand (NT), 610 g kg^−1^ clay, 220 g kg^−1^ silt, 170 g kg^−1^ sand (CT), 580 g kg^−1^ clay, 260 g kg^−1^ silt, 160 g kg^−1^ sand (MT), and 590 g kg^−1^ clay, 340 g kg^−1^ silt, 70 g kg^−1^ sand (F), as previously measured by Pires et al. [30]. According to the USDA soil texture classification system, the soil is considered as having a clay texture [35].

For this study, 81 sub-volumes (selected inside the core scanned samples) were investigated and divided into no application of W-D cycles (F: 7, CT: 9, MT: 10, NT: 11 samples) and application of W-D cycles (F: 11, CT: 11, MT: 10, NT: 12 samples). The differences in the number of samples among treatments (W-D cycles) were due to sample damage caused by the application of the wetting cycles.

### 2.2. Application of Wetting and Drying (W-D) Cycles

After the sampling procedure, the soil samples were wrapped in plastic wrap and carefully placed in cardboard boxes for better transport to the laboratory. The soil sample excess, outside the cylinders, was trimmed in the laboratory using a sharp knife. Afterward, the samples were saturated by the traditional capillary rise method. The wetting (W) procedure consisted in soaking the samples in a tray with the water level just below the top of the steel cylinders. This procedure was carefully carried out for two days (small amounts of water poured into the tray at each hour) to allow the sample’s saturation and to avoid the presence of entrapped air bubbles, which can cause soil aggregate’s slacking [36].

When saturated, the samples were placed on a suction table (EijKelkamp, model 08.01 SandBox) and submitted to a matric potential of −6 kPa until the thermodynamic equilibrium (approximately 3–4 days) was reached [36]. Subsequently, samples were subjected to a new saturation to evaluate possible changes in their structures following sequences of W-D cycles [37,38]. Samples not submitted to any W-D cycle (0 W-D) were considered a reference. After the cycles (12 W-D), the samples were oven-dried at 40 °C for several days to determine their dry masses. The procedure of oven-drying was carried out at temperatures lower than conventional (105 °C) to avoid changes in the soil pore system due to sample shrinkage (clay soil).

### 2.3. Step of Acquisition and Processing of Microtomographic (μCT) Images 

The samples underwent non-destructive 3D X-ray imaging using a GE v|tomex|m μCT system (GE Measurement and Control Solutions, Wunstorf, Germany) at the Hounsfield Facility (the University of Nottingham, Sutton Bonington Campus, Leicestershire, UK). Each sample was scanned for around 10 min at 180 kV voltage and 160 mA current intensity. A 0.1 mm thick copper filter was utilized to eliminate low-frequency X-rays and to reduce beam-hardening artifacts. The exposure time per radiograph was 250 ms.

Images were reconstructed in 32-bit grayscale, aiming to avoid overlapping gray tones. The analysis was performed on images cropped far from the sample edges to minimize possible border effects caused by the sampling procedure. The image pixel size was 35 μm. The reconstruction of the images was carried out using the software provided by the GE manufacturer.

After image reconstruction, a volume of interest (VOI) of 15.1 × 15.1 × 30.1 mm^3^ (430 × 430 × 860 voxels) was selected using the VG StudioMAX^®^ 2.0 software. First, a 3D median filter with a radius of two voxels was applied, followed by an unsharp mask with a standard deviation of one voxel and a weight of 0.8 to reduce noise and artifacts. The last procedure was employed to better discriminate the image’s pore and the solid phases. Next, the grayscale images were segmented using the Otsu non-parameterized built-in procedure (ImageJ 1.42 program). Finally, the images were binarized: white (value 1) corresponding to pores and black (value 0) to soil particles.

### 2.4. Calculation of 3D Lacunarity and Multifractal Parameters

The 3D lacunarity determinations were made using Equation (1) with cubic box sizes of 1, 2, 5, 10, 43, 83, 215, and 430 [39,40,41]. The linear behaviors of the 3D lacunarity curves were investigated in two divisions of intervals: 1 to 10 voxels (1st part) and 43 to 430 voxels (2nd part). Linear adjustments were performed considering determination coefficients (r^2^) greater than 0.95 [25,41].

(1) $$\Lambda \left(\varepsilon \right)=\frac{\sum_{s}s^{2}P\left(s,\varepsilon \right)}{{\left[\sum_{s}\mathrm{sP}\left(s,\varepsilon \right)\right]}^{2}}\;,$$

where s is the number of pixels in black or white colors contained within the box, ε is the box size, and P (s,ε) are the distribution probabilities.

The first-order derivative of the lacunarity (Equation (2)) was used for analyzing the results [42].

(2) $$\frac{d\ln\Lambda \left(\varepsilon \right)}{d\ln\varepsilon }=\frac{\ln\Lambda \left(\varepsilon_{i+1}\right)-\ln\Lambda \left(\varepsilon_{i-1}\right)}{\ln\varepsilon_{i+1}-\ln\varepsilon_{i-1}}\;,$$

where Λ(ε) represents the lacunarity and i indicates each slope point on the lacunarity curve.

The 3D multifractal analysis was carried out by analyzing the multifractal spectrum, as shown in Equations (3) and (4) [43,44,45].

(3) $$f\left(\alpha \left(q\right)\right)=\underset{\varepsilon \to 0}{\lim}\frac{\sum_{i}^{}\mu_{i}\left(q,\varepsilon \right)\log[\mu_{i}\left(q,\varepsilon \right)]}{\log\varepsilon }\;,$$

(4) $$\alpha \left(q\right)=\underset{\varepsilon \to 0}{\lim}\frac{\sum_{i}^{}\mu_{i}\left(q,\varepsilon \right)\log[P_{i}\left(\varepsilon \right)]}{\log\varepsilon }\;,$$

where μ_i_ is the normalized measurement or partition function, q is the statistical moment of the distribution, P_i_(ε) is the pore probability in the ε, α is the internal energy of the system, and f(α) represents the entropy of the system [22].

Parameters related to the degree of multifractality (Δ) and asymmetry (A) were also determined as follows (Equations (5) and (6)) [21]:

(5) $$\Delta =\alpha_{\mathrm{maximum}}-\alpha_{\mathrm{minimum}}\;,$$

(6) $$A=\frac{\alpha_{0}-\alpha_{\mathrm{minimum}}}{\alpha_{\mathrm{maximum}}-\alpha_{0}}\;,$$

where α_maximum_ and α_minimum_ are the Lipschitz–Hölder exponents or singularity multifractal spectrum maximum and minimum, respectively, and α_0_ is the value of the Lipschitz–Hölder exponent at q = 0.

The Δ parameter indicates the degree of heterogeneity of an investigated system. Higher values of Δ indicate more heterogeneous systems and vice versa. Values of A > 1 indicate asymmetry in the left side of the spectrum (pore system), while positive values of A < 1 indicate asymmetries to the other side of the spectrum (solid region). Finally, when A = 1, we have a symmetrical spectrum (equal amounts of pore and solid regions).

The generalized fractal dimension (D_q_), which is related to the moment orders (q’s), was calculated using Equation (7) [43,44].

(7) $$D_{q}=\frac{1}{q-1}\underset{\varepsilon \to 0}{\lim}\frac{\sum_{i}^{}\log\mu_{i}\left(q,\varepsilon \right)}{\log\varepsilon }\;.$$

### 2.5. 3D Geometric Parameters of the Porous System

The 3D geometric parameters of pore connectivity, degree of anisotropy, and tortuosity were used in addition to the multifractal parameters mentioned earlier.

The pore connectivity (C) (Equation (8)) was obtained through the Euler characteristic (EC) (Equation (9)) [30,46,47]. The BoneJ plugin for ImageJ-Fiji software (1.52) was utilized to estimate the degree of connectivity of the soil pore system as follows:

(8) C=1−EC ,

(9) $$\mathrm{EC}=n_{v}-C_{v}\;,$$

where n_v_ is the number of disconnected parts of the porous space per volume and C_v_ is the connectivity by volume (430 × 430 × 860 voxels). The EC indicates how connected a pore is; more negative values of EC represent more connected pore spaces.

The pore tortuosity (τ) (Equation (10)) was calculated by the ratio between the geodesic distance (L_G_) between two connected points in the pore network and the Euclidean distance (L_E_) between them [29]. The OsteoImage software was utilized for this calculation [46,47].

(10) $$\tau =\frac{L_{G}}{L_{E}}\;.$$

The degree of anisotropy (DA) (Equation (11)), which is a measure of how pore substructures are arranged, aligned, and oriented to the selected volume of interest, was calculated as follows [48]:

(11) $$\mathrm{DA}=1-\frac{{\mathrm{Eigenvalue}}_{\mathrm{minimum}}}{{\mathrm{Eigenvalue}}_{\mathrm{maximum}}}\;,$$

where the minimum and maximum eigenvalues refer to the minimum and maximum radii of fitted ellipsoids (x, y, z), respectively.

### 2.6. 3D Normalized Shannon’s Entropy for Pore Space Characterization

Three-dimensional multifractal spectra and normalized Shannon’s entropies were determined using the Non-linear Analysis Scaling System software (NASS—AgriEntech Ltda./Rutgers University, São Carlos, SP, Brazil) [44,49]. The cubic box sizes used in the acquisition of the spectra were the same as those employed to obtain the 3D lacunarities. Moments of distribution (q’s) varying from −0.8 to 1.4 with 0.1 intervals were employed.

The 3D normalized Shannon’s entropy is a measure of uncertainty or imprecision regarding information for a given set of elements as a function of scale or box size. For this study, in which the pore system is composed mainly of two distinct phases (solid and pores), the Shannon’s (H(ε)) and normalized Shannon’s (H*(ε)) entropies were determined using Equations (12)–(14) [49,50,51].

(12) $$H\left(\varepsilon \right)=-\sum_{i=0}^{\varepsilon^{2}}P_{i}\left(\varepsilon \right){\mathrm{logP}}_{i}\left(\varepsilon \right)\;,$$

(13) $$H^{*}\left(\varepsilon \right)=\frac{H\left(\varepsilon \right)}{H_{M}\left(\varepsilon \right)}=-\frac{1}{\log\left(\varepsilon^{2}+1\right)}\sum_{i=0}^{\varepsilon^{2}}P_{i}\left(\varepsilon \right){\mathrm{logP}}_{i}\left(\varepsilon \right)\;,$$

where the term H_M_(ε) = log(ε^2^ + 1) refers to Shannon’s maximum entropy within a cell or binary image with an area of size ε^2^.

In the 3D case, the normalized entropy, given by Equation (13), can be rewritten considering the longitudinal axis equal to the dimensions of the binary image and, therefore, we have:

(14) $$H^{*}\left(\varepsilon \right)=\frac{H\left(\varepsilon \right)}{H_{M}\left(\varepsilon \right)}=-\frac{1}{\log\left(\varepsilon^{3}+1\right)}\sum_{i=0}^{\varepsilon^{3}}P_{i}\left(\varepsilon \right){\mathrm{logP}}_{i}\left(\varepsilon \right)\;.$$

### 2.7. Statistical Analysis

Statistical analysis was performed using the PAleontological STatistics (PAST) software version 3.21 (Oslo, Norway) [52]. Before the statistical analysis, all data were tested to verify their normality and homoscedasticity. Following these previous analyses, the analysis of variance (ANOVA) was employed for verifying statistical differences between the results of f(α_maximum_), α_maximum_, Δ, A, D_0_, D_1_, D_2_, C, τ, and DA. When differences were found by using the ANOVA test (*p* < 0.05), the means were compared through Tukey’s post hoc test (*p* < 0.05).

## 3. Results

### 3.1. 3D Lacunarity

**Figure 1:**
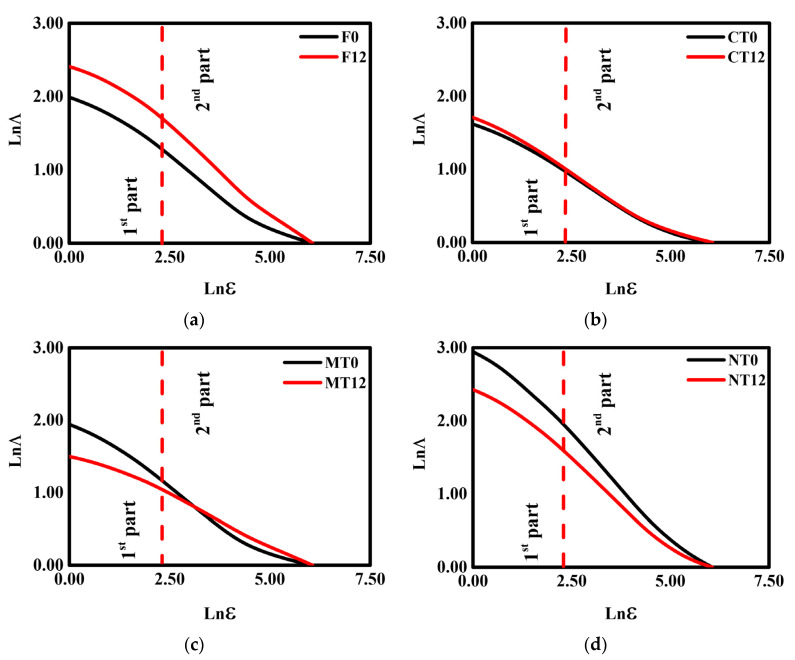
3D lacunarities (Λ) of the samples submitted to 0 and 12 wetting and drying (W-D) cycles. (**a**) Secondary forest—F; (**b**) conventional tillage—CT; (**c**) minimum tillage—MT; (**d**) no tillage—NT. The divisions into two parts (first and second) were made based on the regions where the best linear adjustments occurred in the curves. ε is the box size.

**Figure 2:**
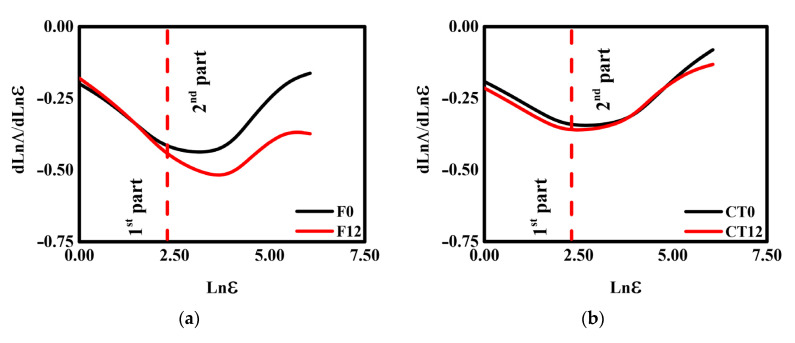

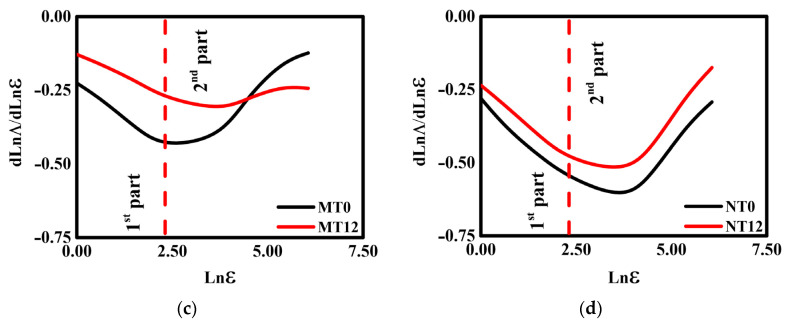
First derivative (dLnΛdLnε) of the 3D lacunarities of the samples submitted to 0 and 12 wetting and drying (W-D) cycles. (**a**) Secondary forest—F; (**b**) conventional tillage—CT; (**c**) minimum tillage—MT; (**d**) no tillage—NT. ε is the box size.

The lacunarity curves with their respective first derivatives for the contrasting management practices and secondary forest samples submitted to the W-D cycles are presented in Figure 1 and Figure 2.

The results indicate that the applications of W-D cycles caused considerable changes in the pore system of F, MT, and NT. The lacunarity for F considerably increased by 33% (Figure 1a), which was followed by a decrease in the soil porosity after the cycles [38]. On the other hand, NT showed a lacunarity reduction of 23% (Figure 1d) followed by an increase in the soil porosity found for this management [38]. The lacunarity for MT (Figure 1c) was characterized by a different behavior when compared to the other managements, with an increase happening from box sizes larger than 43 (above 1.50^3^ mm^3^—second part (dashed line): inversion point). No differences between W-D cycles were noticed for CT (Figure 1b).

The values of the coefficient of determination (r^2^) obtained for the 3D lacunarity curves are presented in Table 1. High values of r^2^ (r^2^ ≈ 1) found for the lacunarity data confirm the quality of the adjustments, which indicates the representativeness of the 3D lacunarity analysis [27,53,54]. For F and NT (0 and 12 W-D), the inflection points occurred at box size of 43 (1.50^3^ mm^3^—second part) (Figure 2a,d). For F, 12 W-D cycles lowered the inflection point values by around 21% (Figure 2a: from −0.45 to −0.55), while for NT (Figure 2d: from −0.63 to −0.53), there was an increase of approximately 15%.

According to Roy et al. [42], the derivative of the lacunarity results reveals the variations in cluster spatial distributions concerning the investigated scale. For F, we found a decrease in the amplitude of pore clusters (amount of pores) around the average diameter of 200 μm. NT experienced an increase in pore clusters with an average diameter > 200 μm [38]. On the other hand, MT exhibited different behavior in its lacunarity and derivative curves (Figure 2c), presenting a displacement of the lower inflection points (from −0.44 to −0.32—an increase of 28%). Such behavior might be possibly associated with the appearance of a greater diversity of clusters of large pores in the size range between 350 and 2800 μm, as shown in Oliveira et al. [38]. For CT (Figure 2b), the derivative curves were similar and close to each other, as the inflections occurred at the same box size (10 = 0.35^3^ mm^3^). The derivative had inflection point values of −0.35 (0 W-D) and −0.37 (12 W-D) (a difference around 5%). These results indicate that applying the W-D cycles did not promote substantial modifications in the pore spatial distributions for CT.

### 3.2. 3D Multifractal Spectra Jointly with Multifractal Parameters and Generalized Multifractal Dimensions

**Figure 3:**
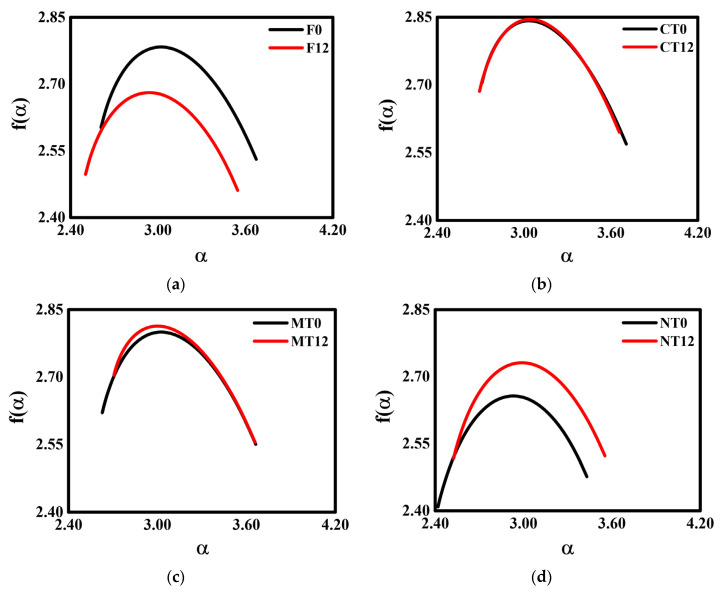
Multifractal spectra (f(α) versus α) of the samples subjected to 0 and 12 wetting and drying (W-D) cycles. (**a**) Secondary forest—F; (**b**) conventional tillage—CT; (**c**) minimum tillage—MT; (**d**) no tillage—NT.

The multifractal spectra (f(α) versus α) for F and the contrasting management practices (CT, MT, and NT) are presented in Figure 3.

The multifractal spectra exhibited slight asymmetry similarities in the curve shapes among managements and the secondary forest, making it possible to divide them into two distinct groups. The results indicated asymmetries to the right side of the spectrum for both 0 and 12 W-D cycles. This behavior was especially noticed for F, CT, and MT. After 12 W-D cycles, F presented an increase of 16% in A (asymmetry parameter) (Table 2 and Figure 3a), while for MT and NT (Figure 3c,d), reductions of 29% and 20% were found, respectively. It implies modifications in the curve shapes, making them less asymmetrical (or more symmetrical—left tendency) by the application of W-D cycles. For CT (Table 2 and Figure 3b), no changes in A were noticed, demonstrating that the cycles did not cause alterations in the asymmetry of the spectra.

Except for MT, the parameter Δ did not exhibit significant changes after the application of the W-D cycles among the contrasting management practices and F (Table 2). The obtained results for Δ indicate that the soil structure exhibited a moderate degree of multifractality for the different managements. It is essential to highlight that Δ is a parameter that can reveal the heterogeneity and complexity of the pore system [21,26,43].

Analyzing the parameters α_maximum_ and f(α_maximum_), which gives one an idea of the average internal energy and entropy of the system under study, it was found that F and NT (Table 2) had variations of around 3% to 4% in their internal energies, suggesting changes in the stability of the soil pore system followed by the W-D cycles. For CT and MT (Table 2), the W-D cycles did not promote changes in the average internal energies of these pore systems, which suggests a certain level of stability of these structures [55].

In a first approach, we might affirm the existence of multifractality of the pore spaces for all the management practices and secondary forest samples before and after the W-D cycles. This fact is corroborated when the generalized fractal dimension is analyzed: D_0_ > D_1_ > D_2_ (Table 2) [23,45,56]. D_0_ was reduced by around 4% for F and increased by around 3% for NT (Table 2). For D_1_, which is related to the information dimension or Shannon’s entropy, a reduction of around 4% was found after applying 12 W-D cycles for F (Table 2). For NT and MT, the opposite behavior was observed, with increases of around 4% and 2%, respectively. These variations in the generalized fractal dimensions may be related directly to changes in widths and heights (frequency distribution amplitudes) of the particle or pore size distribution curves, as reported by Wang et al. [57] and Oliveira et al. [38]. Thus, the value of D_1_ for F indicates that this is the system with the lowest diversity of pores in its porous network. At the same time, for MT and NT, the increase in this parameter would be associated with greater diversity and complexity of the pores [43,44].

The dimension D_2_, which correlates the measures within a range of volumes ε^3^, was changed in F (reduction of 4%), MT (increased 3%), and NT (increased 4%) after the W-D cycles. These D_2_ variations can be correlated with changes in the sample porosities and alterations in the pore’s spatial distributions [23,38,44,57]. Analyzing and comparing our results with those obtained by Oliveira et al. [38], the decrease in the correlation dimension is accompanied by a decrease in the porosity and variability in the size of pore diameters. The opposite relationship is also valid (increase in D_2_) since the same authors found increases in porosity and specific classes of pore sizes for MT and NT. Lee and Lee [51] have reported a linear relationship between porosity and the generalized fractal dimension.

### 3.3. 3D Geometric Parameters

The 3D geometric parameters measured for the contrasting management practices and secondary forest samples studied are presented in Table 3.

The DA for CT, NT, and F did not suffer significant changes with the W-D cycles. Even after successive W-D cycles, the pore system network remained almost isotropic (values close to zero). However, significant changes were observed in C values after the cycles for F and NT (Table 3). It can be seen that the successive W-D cycles caused reductions of around 47% and 13% in the C value for F and MT. On the other hand, the C value for NT increased approximately 26% after the W-D cycles. The results found for C and DA can be linked to variations in porosity (decrease for F and increase for NT) in the soil after the W-D cycles, as demonstrated by Oliveira et al. [38]. Finally, we also observed that τ changed due to the application of W-D cycles for F, NT, and CT (Table 3). An increase of about 11% was noticed for F. On the other hand, τ was reduced by approximately 13% for NT, and no significant changes were observed for the other two management practices (CT and MT).

### 3.4. 3D Normalized Shannon’s Entropy

**Figure 4:**
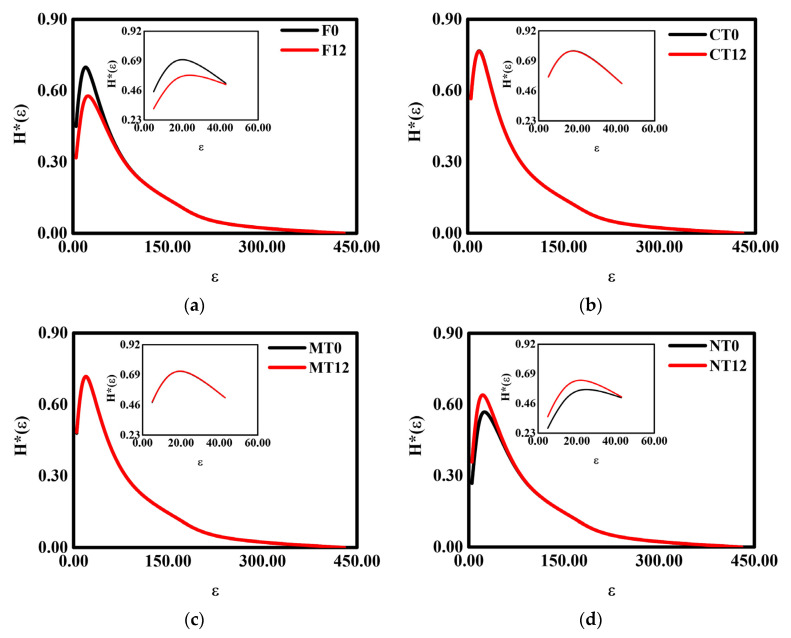
The 3D normalized Shannon’s entropy (H*(ε)) of the samples submitted to 0 and 12 wetting and drying (W-D) cycles. (**a**) Secondary forest—F; (**b**) conventional tillage—CT; (**c**) minimum tillage—MT; (**d**) no tillage—NT. The smaller graphs represent a zoomed-in region with the most significant variation (F and NT) between the curves. ε is the box size.

The 3D normalized Shannon’s entropy (Figure 4) and the multifractal spectra (Figure 3) presented similarities for all the studied treatments. The W-D cycles did not promote significant changes in the 3D normalized Shannon’s entropy for CT and MT (Figure 4b,c). The entropy results of the information dimension (related to porous geometric characteristics) and of the physical–statistical character of the multifractal spectrum f(α_maximum_) (related to the Boltzmann’s entropy [22]) were similar between F and NT, mainly for D_1_. The parameters D_1_ and f(α_maximum_) decreased for F and increased for NT after W-D cycles, revealing alterations in the heterogeneity of the pore systems. For F, the application of the cycles demonstrated a more stable soil pore system (less diversification of pores with different characteristic sizes in the porous network). The opposite was noticed for NT, where an increase in the soil pore system heterogeneity and growth of the pore sizes was noticed [38].

**Figure 5:**
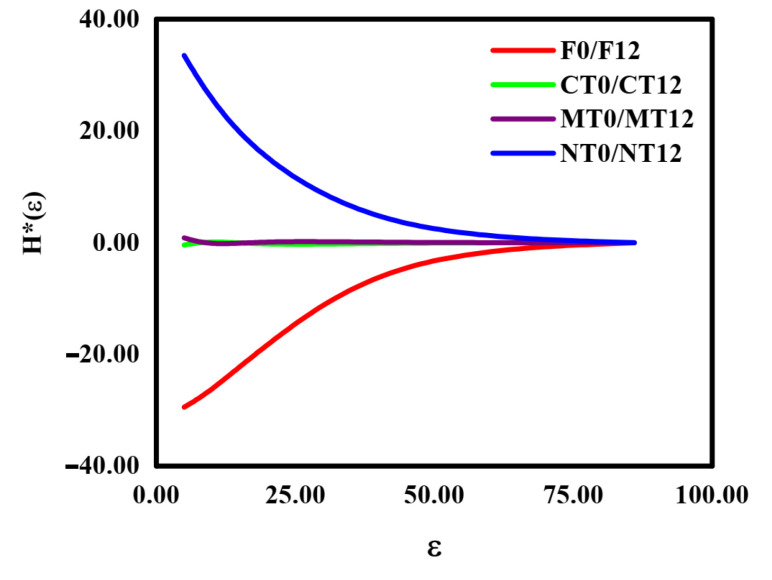
Variation of the 3D normalized Shannon’s entropy (H*(ε)) of the samples submitted to 0 and 12 wetting and drying (W-D) cycles. Secondary forest—F; conventional tillage—CT; minimum tillage—MT; no tillage—NT. ε is the box size.

The observed variations in H*_maximum_(ε) for NT and F occurred in the box size of 20 (0.70^3^ mm^3^) after the W-D cycles. For NT (Figure 4d), there was an increase in the Shannon’s entropy of approximately 14%. It implies greater randomness in soil properties such as pore shape and size distributions, and pore functionality. The size of pores significantly impacts their functionalities, affecting the air movement and water drainage in transmission pores, retention of water in storage pores, and retention and diffusion of chemical components in residual pores [35]. For F (Figure 4a), H*_maximum_(ε) was reduced by around 18%, which means a homogenization of the distribution of pores in the soil pore system, as mentioned earlier [49,57]. The effects of W-D cycles on 3D normalized Shannon’s entropy can be better evaluated by inspecting Figure 5. Considering the studied soil’s two phases (particles and pores), the uncertainty in the detection or accounting of pores for small box sizes is considerably lower, whereas, for large box sizes, the uncertainties increase, presenting a low probability to detect a pore on its completeness. As seen in Figure 5, the W-D cycles were more effective (generated more significant changes) on smaller box sizes for F and NT, occasioning a lesser degree of pore disorder in these box sizes.

We observed that the modifications caused by the W-D cycles in the 3D normalized Shannon’s entropy were abruptly attenuated up to a specific maximum box size (Figure 5). It happened because the effect of the cycles becomes insignificant due to the low pore occupancy rate (amount or numbers of pores) for larger volumes. Regarding CT and MT, there was no variation in the 3D Shannon’s entropy due to the cycles since their accessible states *i* (pores or solid particles) do not have the same probability of occurrence. In the previous specific case (CT and MT), the complexity of the porous system remained the same (did not change relatively) after the W-D cycles [49,50].

## 4. Discussion

### 4.1. 3D Lacunarity

In this study, the effect of W-D cycles in the pore system complexity of a Rhodic Hapludox submitted to contrasting management practices was investigated. A 3D multifractal analysis, entropy, and lacunarity were employed to characterize the soil pore spaces before and after the W-D cycles.

The lacunarity, which reflects the degree of spatial distribution patterns of the pores (pore clusters) inside the soil [27,54], exhibited contrasting results among managements (Figure 1 and Figure 2). Lacunarity value increases are associated with increases in the pore clusters’ degree of dispersity in porous systems. Thus, the analysis of the lacunarity indicates that W-D cycles induced changes in the heterogeneity and dispersity of the soil pore system clusters for F, MT, and NT. The soil under the secondary forest presented an increase in the dispersity of pore clusters after applying the cycles, while the opposite was associated with MT and NT. The presence of organic materials in high amounts in the soil under forests and the absence of any management can induce significant changes in its macropore fraction under W-D cycles, impacting the stability of the pores and also the water retention characteristics [2,38]. Although soils under NT and MT are characterized as being rich in organic materials (mainly in the topsoil), the decrease in the heterogeneity of the pores following the cycles might be related to changes in the structural porosity associated with possible aggregate coalescence due to the clay nature of the soil studied [58,59] and increases in the proportion of large-sized pores (> 500 μm) as observed by Oliveira et al. [38].

Conventional tillage, the other management practice studied, is characterized by the disruption of the topsoil due to plowing and harrowing operations employed to turn over and loosen the top soil layer. Surprisingly, this management did not present changes in the pore heterogeneity due to the W-D cycles, which indicates that soil under CT presented a more stable structure. However, the breaking of the aggregates caused by the plowing and harrowing operations usually affects the organic matter content and oxide distribution in the soil under CT. Therefore, decreases in organic matter can have critical reflections on the way the soil submitted to this management can respond to W-D cycles [2,3,60,61,62,63].

### 4.2. 3D Multifractal Spectra with Quantified Parameters and Generalized Multifractal Dimensions

The 3D multifractal and asymmetry analyses showed remarkable results on the patterns of the spatial variability of the studied soil pore systems (Figure 3). The asymmetry parameter (A), when positive and smaller than 1 (Table 2), gives an idea about the prevalence of high singularity exponents α [64]. The asymmetry for the right side of the spectrum observed for all the samples indicates low patterns of spatial variability, and here it was associated with low spatial variability of the pore size distribution [44]. Thus, this investigation showed that for F, MT, and NT, the observed asymmetries could be linked to less spatial variability in the range of pore sizes investigated; while for CT, smaller changes in the spectra indicated small or minor changes in the heterogeneity of the pore size distribution [56].

The wetting and drying cycles promoted f(α_maximum_) changes for F and NT (Table 2). Physically, this indicates that there were variations in the entropies of the soil pore system for these managements. Forest samples presented a reduction of about 4% in the global entropy, indicating that the W-D cycles contributed to homogenizing the pore distribution, i.e., lower pore size diversity [38]. For NT, the global entropy increased by about 3%. This result is characteristic of an enlargement of the pore distribution range and the degree of disorder for this management. The W-D cycles did not produce variations in the global entropies of CT and MT. The analysis of the generalized fractal dimension also demonstrated that all managements maintained their multifractality even after the W-D cycles (Table 2). The high D_0_ (capacity dimension) values found are a strong indication of a high degree of information or a broader range of pore diameters in the investigated samples [65,66]. We also observed that 12 W-D cycles did not promote significant changes in D_0_ for MT and CT, suggesting that even after the cycles, the soils under these managements maintained the degree of information related to their pore size distributions.

### 4.3. 3D Geometric Parameters and 3D Normalized Shannon’s Entropy

The morphological parameters (Table 3) show that the pore connectivity was negatively affected by the W-D cycles for F. Oliveira et al. [38] found at the same experimental site that the W-D cycles reduced the proportion of transmission pores in F, which can explain the results of C observed in our study. Changes in the proportions of cracks and channels between aggregates can significantly impact inter-connected pores, as demonstrated by Peña-Sancho et al. [67]. No-tillage samples presented increased pore connectivity after applying the cycles. It is probably associated with an increase in the number of transmission pores [38]. The maintenance of crop residues after harvesting at the topsoil is crucial to preserving the pores associated with water transport in NT systems [68,69]. The results of pore tortuosity showed an increase for F, which suggests a certain degree of misalignment (high sinuosity) of the pores. The increase in τ can also explain the decrease in C observed in F. The decrease in the soil heterogeneity (lacunarity results) might explain the tortuosity results found here for NT, which was characterized by more aligned pores after the W-D cycles.

Regarding the 3D Shannon’s entropy (Figure 4), the most critical changes in the soil pore systems induced by the W-D cycles were observed for F and NT. Lee and Lee [51] indicated that Shannon’s entropy could be directly related to the porosity of the samples. More specifically, larger values of this parameter indicate samples with large porosities. Thus, the decrease in H*_maximum_(ε) and H*(ε) noticed for F and the increase for NT were associated with the variations in the soil porosity (decreased for F and increased for NT) observed for these two treatments after the applications of the W-D cycles [51]. The same linear relationship with porosity was not verified for CT and MT since no changes were observed with the cycle application in the parameters H*(ε) and H*_maximum_(ε).

Finally, the results of this study demonstrate that the 3D multifractal and lacunarity analyses are useful tools to characterize the soil pore system dynamics under sequences of W-D cycles. Combining these techniques with the morphological analysis of the soil structure can provide a better investigation of the changes in the complexity of soil pores and the impact of the cycles on their functions. It was found that the 3D multifractal approach in association with lacunarity and geometric parameters was able to identify, quantify, and characterize the changes caused in the complexity of the pore system of samples submitted to W-D cycles.

## 5. Conclusions

The wetting and drying cycles caused noticeable changes in the 3D lacunarity properties of F, MT, and NT. The modifications in 3D lacunarities and their inflection points revealed alterations in the porosities and spatial distribution of the pores. The 3D multifractal spectra provided detailed analyses of the changes in the pore system of the management practices investigated by the action of W-D cycles, making it possible to observe tendencies in the behavior of the soil pore network in terms of asymmetry (multifractal spectrum), especially for F and NT.

Multifractal spectra indicated low spatial variability of pore sizes in F, increasing this variability for MT and NT. Multifractal parameters α_maximum_ and f(α_maximum_), which reflect thermodynamic properties, exhibited significant differences for F and NT. These differences were associated with the heterogeneity of the pore systems of the soils under these managements, which reflects the diversity of the pore size distribution and degree of disorder found for F and NT. The sequence order found for the generalized 3D fractal dimensions (D_0_ > D_1_ > D_2_), following the W-D cycles, revealed the strong multifractality of the pore systems analyzed. The generalized fractal dimensions D_1_ and D_2_ for F, MT, and NT indicated alterations in the heterogeneity patterns of their pore size distributions after the cycles.

The parameters C and τ and the 3D normalized Shannon’s entropy confirmed that F and NT were the treatments that suffered the most significant modifications by the application of W-D cycles. Lower C values, especially for F, indicated the existence of isolated pores, possibly inaccessible to water. On the other hand, higher C values found in NT are characteristic of a pore system that is more favorable to water infiltration and redistribution as expected for this management practice. The differences in τ for F and NT showed that these treatments had their pore sinuosities influenced by the W-D cycles, with possible influence on the continuity of the pores as observed by the found C values.

In summary, the multifractal approach used here pointed out that the porous environments of F and NT showed similar variations (most of the multifractal parameters quantified) with the W-D cycles. On the other hand, the pore system of the samples under CT and MT had similarities among some physical properties, as in the case of the multifractal parameters that did not show considerable changes after the W-D cycles.

## Figures and Tables

**Table 1:** Values of the coefficient of determination (r^2^) of the linear adjustments for the two parts (first and second) of the 3D lacunarity (Λ) curves for the areas under secondary forest (F) and the three management practices (conventional tillage—CT, minimum tillage—MT, and no tillage—NT) submitted to 0 and 12 wetting and drying (W-D) cycles.

	Coefficient of Determination (r^2^)	
Management	First Part	Second Part
F0	0.98	0.96
F12	0.98	0.99
CT0	0.99	0.97
CT12	0.99	0.97
MT0	0.99	0.96
MT12	0.99	0.99
NT0	0.99	0.98
NT12	0.99	0.96

**Table 2:** Multifractal parameters calculated from the multifractal spectra curves (f(α) versus α) for the secondary forest (F) and the three management practices (conventional tillage—CT, minimum tillage—MT, and no tillage—NT) submitted to 0 and 12 wetting and drying (W-D) cycles.

Management	∆	A	α_maximum_	f(α_maximum_)
F0	1.07 ± 0.05	0.62 ± 0.05 ^α^	3.67 ± 0.04 ^α^	2.78 ± 0.01 ^α^
F12	1.04 ± 0.02	0.72 ± 0.05 ^β^	3.55 ± 0.05 ^β^	2.68 ± 0.02 ^β^
CT0	1.00 ± 0.06	0.49 ± 0.09	3.71 ± 0.08	2.84 ± 0.02
CT12	0.97 ± 0.04	0.57 ± 0.10	3.66 ± 0.05	2.85 ± 0.02
MT0	1.04 ± 0.05 ^α^	0.63 ± 0.08 ^α^	3.66 ± 0.06	2.80 ± 0.03
MT12	0.95 ± 0.09 ^β^	0.45 ± 0.11 ^β^	3.66 ± 0.06	2.81 ± 0.02
NT0	1.01 ± 0.05	1.03 ± 0.10 ^α^	3.43 ± 0.07 ^α^	2.66 ± 0.03 ^α^
NT12	1.03 ± 0.06	0.83 ± 0.08 ^β^	3.55 ± 0.05 ^β^	2.73 ± 0.02 ^β^
**Management**		**D_0_**	**D_1_**	**D_2_**
F0		2.78 ± 0.01 ^α^	2.65 ± 0.02 ^α^	2.61 ± 0.03 ^α^
F12		2.68 ± 0.02 ^β^	2.54 ± 0.02 ^β^	2.50 ± 0.03 ^β^
CT0		2.84 ± 0.02	2.74 ± 0.04	2.71 ± 0.04
CT12		2.85 ± 0.02	2.73 ± 0.03	2.69 ± 0.04
MT0		2.80 ± 0.03	2.67 ± 0.03 ^α^	2.62 ± 0.04 ^α^
MT12		2.81 ± 0.02	2.72 ± 0.03 ^β^	2.71 ± 0.04 ^β^
NT0		2.66 ± 0.03 ^α^	2.48 ± 0.03 ^α^	2.41 ± 0.03 ^α^
NT12		2.73 ± 0.02 ^β^	2.57 ± 0.04 ^β^	2.52 ± 0.04 ^β^

Δ—Degree of multifractality; A—Degree of asymmetry; D_0_—Box count dimension or capacity dimension; D_1_—Information dimension; D_2_—Correlation dimension; α_maximum_—Related to internal system energy; f(α_maximum_)—Related to the system global entropy (Boltzmann’s entropy). Results followed by different Greek letters differed statistically from each other by ANOVA and Tukey’s test (*p* < 0.05). Comparisons were made within each management considering W-D cycles.

**Table 3:** Geometric parameters associated with the soil pore system of the secondary forest (F) and the three management practices (conventional tillage—CT, minimum tillage—MT, and no tillage—NT) submitted to 0 and 12 wetting and drying (W-D) cycles.

Management	DA	C	τ
F0	0.21 ± 0.05	2.78 ± 0.30 ^α^	1.46 ± 0.03 ^α^
F12	0.25 ± 0.09	1.48 ± 0.14 ^β^	1.62 ± 0.08 ^β^
CT0	0.31 ± 0.09	4.20 ± 1.49	1.36 ± 0.06
CT12	0.34 ± 0.10	4.36 ± 1.04	1.42 ± 0.09
MT0	0.29 ± 0.10	2.72 ± 0.70	1.47 ± 0.07
MT12	0.36 ± 0.07	2.36 ± 0.98	1.39 ± 0.09
NT0	0.39 ± 0.13	1.18 ± 0.18 ^α^	1.84 ± 0.15 ^α^
NT12	0.31 ± 0.09	1.49 ± 0.19 ^β^	1.59 ± 0.09 ^β^

DA—Degree of anisotropy; C—Pore connectivity; τ—Pore tortuosity. Results followed by different Greek letters differed statistically from each other by ANOVA and Tukey’s test (*p* < 0.05). Comparisons were made within each management considering W-D cycles.

## Data Availability

All data are available upon reasonable request to deoliveirajato@gmail.com.
